# Supplementary material for: Splice-Junction-Based Mapping of Alternative Isoforms in the Human Proteome
Source: Cell Rep. Author manuscript; Available in PMC 2020 Jan 15. (PMC6961840; doi:10.1016/j.celrep.2019.11.026)
Supplement: 3 [file NIHMS1546469-supplement-3.zip › DF2/PXD0010154/Lung-98-Q9H4L5-TYSAPAINAIQVPKPFSGPVR.pdf]

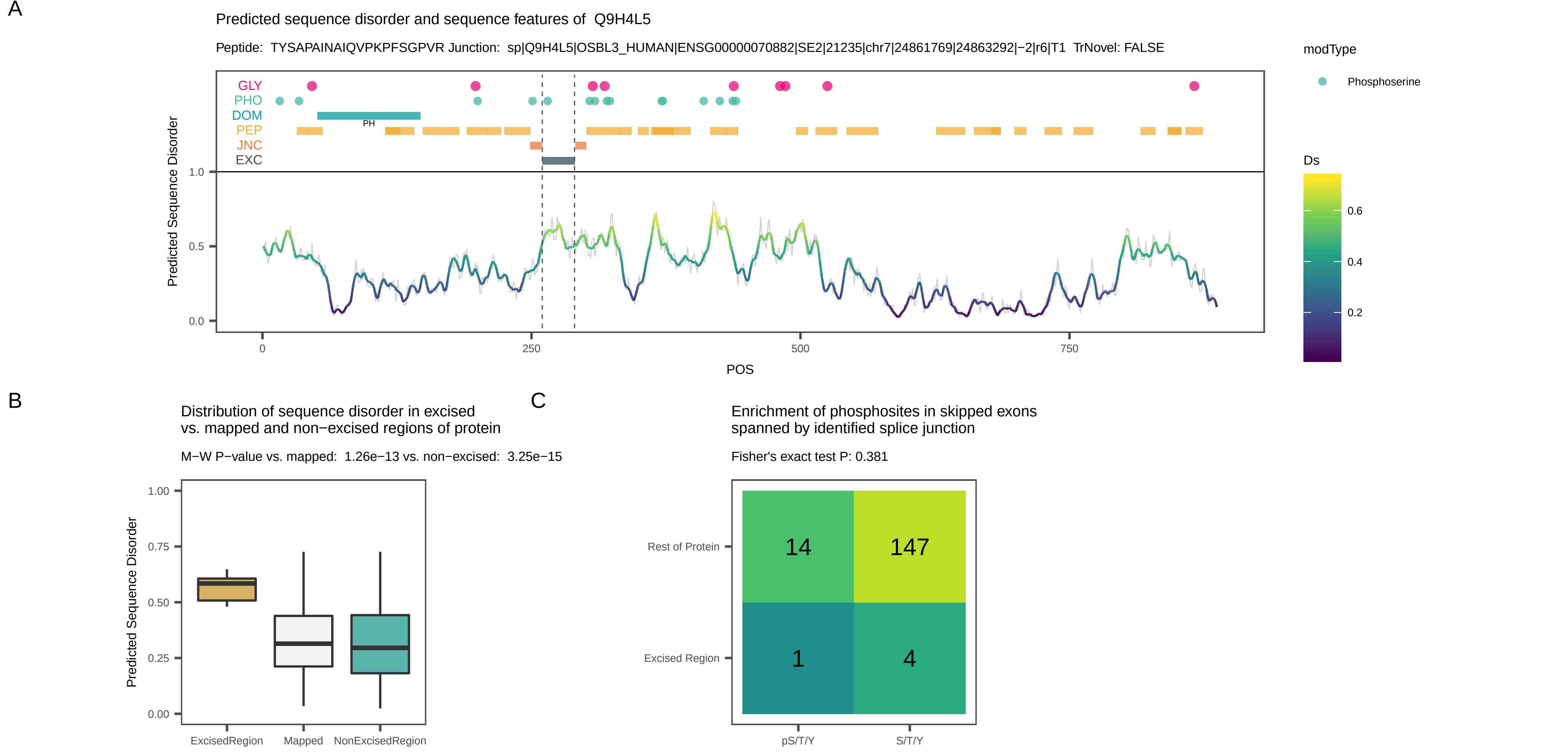
A
Predicted sequence disorder and sequence features of Q9H4L5
Peptide: TYSAPAINAIQVPKPFSGPVR Junction: sp|Q9H4L5|OSBL3_HUMAN|ENSG00000070882|SE2|21235|chr7|24861769|24863292|−2|r6|T1 TrNovel: FALSE
modType
Phosphoserine
GLY
PHO
DOM
PEP
JNC
EXC
PH
Predicted Sequence Disorder
1.0
0.5
0.0
0
250
500
750
POS
Ds
0.6
0.4
0.2
B
Distribution of sequence disorder in excised vs. mapped and non−excised regions of protein
M−W P−value vs. mapped: 1.26e−13 vs. non−excised: 3.25e−15
Predicted Sequence Disorder
1.00
0.75
0.50
0.25
0.00
ExcisedRegion
Mapped
NonExcisedRegion
C
Enrichment of phosphosites in skipped exons spanned by identified splice junction
Fisher's exact test P: 0.381
Rest of Protein
Excised Region
14
147
1
4
pS/T/Y
S/T/Y
